# Persistent Cervical Infection with the Chikungunya Virus

**DOI:** 10.1590/0037-8682-0304-2024

**Published:** 2025-05-09

**Authors:** José Eduardo Pérez-Pérez, Oscar Guzmán-Martínez, Jesús Miguel Torres-Flores, Clara Luz Sampieri, Julio Isael Pérez-Carreón, Nayali López-Balderas, Miguel Ángel Barragán, Guillermo Mendoza-Cervantes, Fabio García-García, Alma Campos, David Sánchez-Marín, Hilda Montero

**Affiliations:** 1 Instituto de Salud Pública, Universidad Veracruzana, Xalapa, Veracruz, México.; 2 Instituto de Ciencias de la Salud, Universidad Veracruzana, Xalapa, Veracruz, México.; 3 Centro de Investigaciones Biomédicas, Universidad Veracruzana, Xalapa, Veracruz, México.; 4 Instituto Politécnico Nacional, Cuidad de México, México.; 5 Instituto Nacional de Medicina Genómica, Cuidad de México, México.; 6 Instituto de Medicina Forense, Universidad Veracruzana, Boca del Río, Veracruz, México.; 7 Hospital de Alta Especialidad de Veracruz, Veracruz, Veracruz, México.; 8 Centro de Investigación en Micología Aplicada, Universidad Veracruzana, Xalapa, Veracruz, México.; 9 Facultad de Medicina, Universidad Nacional Autónoma de México (UNAM), Ciudad de México, México.

**Keywords:** Chikungunya virus, viral persistence, cervix

## Abstract

Chikungunya virus (CHIKV) induces an acute infection known as chikungunya fever; however, the persistence of CHIKV infections has been reported. This report investigated the persistence of CHIKV over six months in the cervix of two infected women. The infectious CHIKV was isolated from three samples. Our findings demonstrated the persistence of CHIKV in the cervix, highlighting the importance of evaluating other tissue types.

## INTRODUCTION

First described in 1952 during an outbreak of febrile illness in Tanzania^1^, Chikungunya virus (CHIKV) is an arthropod-borne virus (arbovirus) belonging to the *Alphavirus* genus of the *Togaviridae* family^2^. Mature CHIKV particles are enveloped, containing a single-stranded RNA genome of approximately 12 kb in length^1^ ^,^ ^2^.

The CHIKV is mainly transmitted through the bite of infected female *Aedes* mosquitoes. This vector, as well as *Aedes aegypti* and *Aedes albopictus* ^3^, has been implicated in the transmission cycle of this virus in urban and peri-urban areas. Viral replication commences in the dermal fibroblasts once an infected mosquito bites a vertebrate host; the virus proliferates before entering the bloodstream. The target cells of CHIKV include monocytes, hepatocytes, chondrocytes, endothelial cells, fibroblasts, and myocytes^2^ ^,^ ^3^ ^,^ ^4^. 

CHIKV infection leads to chikungunya fever, which is symptomatic in 85% of cases. The acute phase of the disease is characterized by the presence of fever, arthralgia/arthritis, fatigue, myalgia, nausea, and vomiting. The development of a generalized maculopapular rash has been reported in some cases^2^. Persistence of CHIKV infections has not been thoroughly characterized. However, several reports have indicated the presence of CHIKV-like symptoms in 30-40% of patients^2^ ^,^ ^4^, highlighting its prevalence in public health systems. The persistence of CHIKV infections years after the first infection has also been reported, with persistence in the bone and joint tissues, the common sites of CHIKV persistence, triggering rheumatoid arthritis and ankylosing spondylitis^3^ ^,^ ^4^.

Although CHIKV is commonly transmitted through the bites of infected mosquitoes, vertical transmission has also been reported^5^. Notably, CHIKV RNA has been detected in the semen and vaginal fluid of infected patients during the acute phase of the disease^4^; however, CHIKV has been persistently detected in semen^6^ ^,^ ^7^ ^,^ ^8^.

The persistence of CHIKV RNA and E2 protein was analyzed in the cervical samples acquired from one asymptomatic woman and one woman with symptoms consistent with chikungunya fever in the present study. The databases of Cochrane Library, LILACS, SciELO, MEDLINE, PubMed, and PubMed Central (PMC) were searched to retrieve publications addressing the persistence of CHIKV in the cervix. However, no such publications could be retrieved, indicating that this is the first study on this subject.

## CASE REPORT

Case 1 was an asymptomatic 60-year-old woman who was the sexual partner of a male patient diagnosed with ZIKV infection through RT-PCR. The patient had no comorbidities or a history of symptoms compatible with arboviruses. The patient was not receiving any medications at the time of the study. Serological analysis revealed negative anti-CHIKV IgM antibodies, but positive anti-CHIKV IgG antibodies. The patient also tested positive for anti-ZIKV IgG antibodies and negative for anti-ZIKV IgM, anti-DENV IgM, and anti-DENV IgG antibodies. 

Case 2 was a symptomatic 26-year-old woman with acute CHIKV infection at the time of enrollment in the study. The patient presented with arthralgia, fever of 39.5 ºC persisting for 2-3 days, loss of appetite, dizziness, and exanthema. CHIKV was detected in the serum and urine samples of this patient through endpoint RT-PCR. The patient had no comorbidities and was not receiving any medication at the time.

Cervical scrapings were obtained longitudinally from both patients at 0, 4, 6, and 10 months. For Case 1, time 0 corresponds to the time-point the patient was enrolled in the study. For case 2, time 0 corresponded to the sample acquired 21 days after the onset of the symptoms of chikungunya fever. No macroscopic lesions were observed during sample collection; only data associated with inflammation were obtained. The patients did not report any gynecological symptoms.

A smear from the cervical scrapings was used to detect CHIKV E2 protein using immunofluorescence assays. The cervical cytobrush was placed in Leibovitz viral transport media (11415056, Gibco, UK) containing antibiotics and an antimycotic after obtaining the smear and kept frozen at -80 ºC for further use in RNA viral detection and viral isolation. 

### Detection of E2 protein of CHIKV in cervix

**FIGURE 1:**
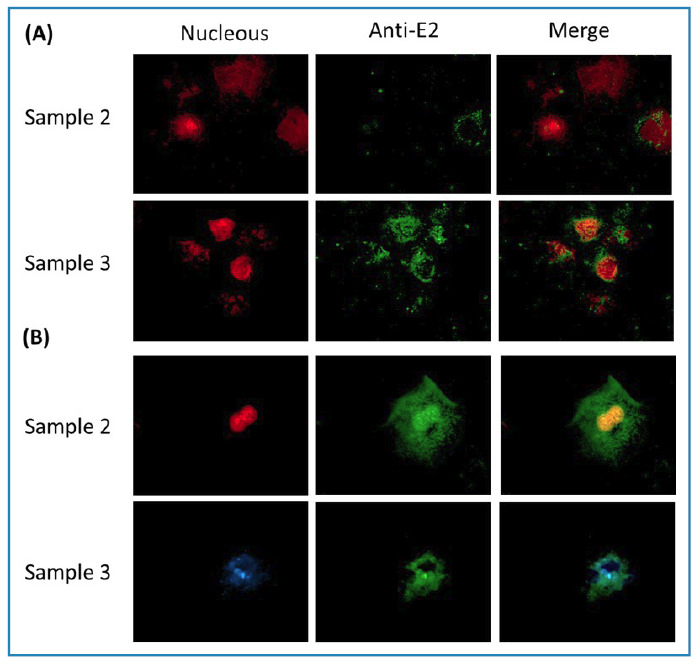
The E2 viral protein of chikungunya virus (CHIKV) detected in the cervical cells. The cells were fixed with reactive-grade acetone at 4°C for 1 minute and stained with an antibody targeting the E2 protein (green). The cellular nuclei were stained with propidium iodide (red) or DAPI (blue). Case 1: asymptomatic patient **(A)**, case 2: symptomatic patient **(B)**. A representative image of a field with cells positive to the detection of E2 is shown (100X magnification).

The E2 protein of CHIKV in all cervical samples (up to the 6-month sample) acquired from the two patients was detected through immunofluorescence assays conducted using an anti-E2 antibody (MAB12130-200; Native Antigen Company, UK). Figure 1 presents a representative image. The 10-month sample could not be analyzed in either case. 

### RNA viral detection

CHIKV RNA was obtained from the total RNA extracted from cervical scraping samples using the QIAamp Viral RNA Mini Kit (52906, QIAGEN, USA) in accordance with the manufacturer’s instructions. Endpoint RT-PCR assays were performed using a One-Step RT-PCR kit (210212; QIAGEN, USA). Real-time RT-PCR was performed using a ZDC Multiplex RT-PCR Assay (12003818, Bio-Rad, USA). 

CHIKV RNA was detected through endpoint RT-PCR in all samples acquired up to the 4-month time-point in case 1. At the same time, viral RNA was detected through real-time RT-PCR in case 2. The time of infection was unknown as Case 1 did not exhibit any signs or symptoms; therefore, the maximum duration of persistence could have been longer. Sanger sequencing of the RT-PCR products was performed using the following CHIKV genome-specific oligonucleotides: forward primer sequence, TAGAGCAGGAAATTGATCCC; and reverse primer sequence, CTTTAATCGCCTGGTGGTGGTAT. Only one sample was sequenced and registered in the NCBI for Biotechnology Information database. Alignment was performed, and a 92% consistency with the CHIKV sequence was identified (Table 1).

**TABLE 1:** Chikungunya virus detected in the cervical tissue.

	Sample number	Time (months)	Endpoint RT-PCR	Smear/ Immunoreactivity	Viral culture RT-PCR	Real-time RT-PCR	Sequency
Case 1 Asymptomatic	1	0	+	NA	-	NA	GenBank PP964031.1
	2	4	+	+	+	NA	NA
	3	6	-	+	+	NA	NA
	4	10	-	NA	-	NA	NA
Case 2 Symptomatic	1	0	+	NA	-	NA	NA
	2	4	-	+	NA	+	NA
	3	6	-	+	+	NA	NA
	4	10	-	NA	-	NA	NA

**RT-PCR:** reverse transcription followed by polymerase chain reaction; **-:** negative; **+:** positive; **NA:** not available.

### Viral isolation

To assess the presence of the infectious virus in the samples, viral isolation procedures were carried out in Vero cells cultured in DMEM supplemented with antibiotics and antimycotics. The samples were lysed through cryofracture after 10 days, and viral RNA was extracted and detected using endpoint RT-PCR. Three samples, two from case 1 (4 and 6 months) and one from case 2 (6 months), were positive for CHIKV RNA after viral isolation, indicating the presence of infectious viral particles in the samples (Table 1). 

## DISCUSSION

No previous study has reported the persistence of CHIKV in the cervix. Thus, the present study is the first study to report that cervical cells represent another location for the persistence of CHIKV. This aspect has remains to be explored and requires attention owing to the possible long-term effects of continuous viral replication, which could be similar to the complications observed in infected bone and joint tissues^4^. Consistent with the findings of the present study, CHIKV possesses ample tropism and that epithelial cells in the cervix express Matrix Remodeling-Associated 8 protein (MXRA8), a known receptor for CHIKV^9^ ^,^ ^10^. 

The CHIKV E2 protein and RNA detected in the present study exhibited variability among the samples. This may be attributed to three different phenomena: 1) the differential distribution of sites of persistent CHIKV replication in the cervix, 2) the loss of viral and cellular material owing to the application of the smear technique, which resulted in the loss of viral RNA in the sample used for RT-PCR detection, and 3) low viral replication. The latter has been observed in other viruses, such as Zika, wherein the viral titer in the cervix was lower than that in the serum and urine samples^11^ ^,^ ^12^.

The detection of infectious viral particles in cervical samples highlights the risk of sexual transmission of CHIKV, as reported for other arboviruses such as the Zika virus or dengue^11^ ^,^ ^13^. This emphasizes the relevance of the findings of the present study and provides a basis for further research using larger samples and longer follow-up periods. Furthermore, the persistence of CHIKV in the cervix could be the cause of vertical transmission^5^ or abortion^14^, as recently reported, requires further investigation.

### Ethical statement

 This study was approved by the research and ethics committees of the corresponding institutions. The study began in July 2018, and both patients signed informed consent forms indicating their agreement to participate.
