# Erratum

**DOI:** 10.1590/0037-8682-0304e-2024

**Published:** 2025-06-27

**Revista da Sociedade Brasileira de Medicina Tropical/Journal of the Brazilian Society of Tropical Medicine**

Title: Persistent Cervical Infection with the Chikungunya Virus 

 Vol.:58 | (e00812-2025) | 2025 | doi: https://doi.org/10.1590/0037-8682-0304-2024

**Author Page 1/1**

 Alma Campos 

**Should read:**

**Alma D. Campos Parra**

